# The Never‐ending Shift: A feminist reflection on living and organizing academic lives during the coronavirus pandemic

**DOI:** 10.1111/gwao.12451

**Published:** 2020-05-05

**Authors:** Ilaria Boncori

**Affiliations:** ^1^ Faculty of Humanities University of Essex UK

**Keywords:** academia, coronavirus, Feminism, flexible working, pandemic

## Abstract

This article offers a feminist reflection written as a nocturnal stream of consciousness exposing the embodied, emotional and professional experience of living and working during a pandemic outbreak. Framed within a feminist approach, this personal narrative provides an example of the effects of such unexpected and unprecedented circumstances on personal and professional academic lives. Developed during the first stage of the (inter)national coronavirus pandemic, my reflections address issues of privilege; emotional labour; the virtual invasion of the home space within the current increasingly ambiguous space of ‘the workplace'; workload; and wellbeing. Further, I consider how the newly enforced flexible work measures based on online tools have turned current work–life dynamics into a ‘Never‐ending Shift'.

## 1 INTRODUCTION

It's 2:13 am. I am rocking in the grey and white chair my mother gave us as a present when my baby was born. A handmade blanked on the side, soft toys peeking out of boxes, a bunny forgotten under a chest of drawers. She's no longer an infant now, I look at her as she fights sleep after waking up from a bad dream in the middle of the night. We've been here for almost 20 minutes, in this strange intimacy of her bedroom illuminated by stars, pollinated by elephants, assembled with the love we have for her. I treasure this time, more than she will ever know. I try to fix every expression, every hug, every funny new word she says in my memory, just in case.

‘*Mamy, I want cuddle*' she pleads with that cute and stern expression she has when half asleep, her sight being caressed into submission. She searches for her milk bottle — this may be a good sign. At this strange hour, it feels like the coronavirus pandemic outbreak may just be a child's nightmare. I am grateful that she doesn't understand what is happening, that to her she is just spending more time at home with her parents, like when we are on those few precious holiday hours filled with adventures and play. She lives her days with cheeky joy; and having to care for her brings both relief and complexity to our days. She shares house chores with daddy, cooks with mamy, plays football every day and gets to do as many puzzles as she wants without time restrictions.

‘*Mamy, where is zeebla? I want zeeeblaaa*' she moans almost tearfully. ‘*Zebra is sleeping in the kitchen. Lay down darling, it's sleepy time*', I respond with what I hope is a soothing voice. At this time of the night my thoughts unravel, galloping out of control around my mind, more genuine and unfiltered by daytime logic. It feels like a mind game, a chain of uncontrolled free associations. My emotions send shock waves on the frayed surface of my consciousness. My many identities and conflicting priorities overlap in waves — daughter who must care from a distance for my elderly and ill parents in Italy; female academic in a quest for professorship; senior leader with responsibility to take forward a plan of action to counteract the negative impact of COVID‐19 on our community and organization; mother who wants to spend quality time with her child; wife who treasures every moment with her partner; homeowner with walls to repair; chef with meals to plan; over‐planner with anxiety to manage. I try to relax; I try to do breathing exercises whilst I rock in the dark, pretending to be asleep in the hope that this will trick my child into Morpheus' embrace; I try to stay positive and count my many blessings; I try in vain to free my mind and my heart. How long will this last? Have we found an alternative for practical assessment in that department? How are we meeting stubborn external accreditation requirements under lockdown? How can we support students in difficult situations? Is the leftover sauce still edible for lunch? Do we have enough nappies in the cupboard? Do we have enough paracetamol to keep her fever down in case she has febrile convulsions again? What time is my first meeting tomorrow? I need to cancel the dentist.

I say to myself that I should not be on social media, that I have to limit my exposure to negativity. I try to spread some positive vibes, so I post pictures of blossoming flowers in my garden and the outcomes of my baking attempts, mementos of a smiley child engaged in everyday adventures and art. But I cannot stop myself from sharing political concerns and pleas for civic responsibility, which bring out differences amongst the people I know personally and at work. Through social media, I am now exposed to their personal beliefs, some of which are selfish, intolerant, racist, elitist and plain unreasonable; these ideas go against every feminist fighting fibre in my body and soul, requiring acceptance and compromise that I am not able or willing to deliver. Maybe the gravity of the situation, my exhaustion and frustration have made me less understanding, and more selective of whom I associate with when I have a choice. I am also less willing and able to compromise on my ideals and over the years I have become more vocal about being a feminist.

The past month is really having an emotional toll on me. I need some time to recover, to do nothing, to read and exclude myself from the world. This forced isolation is actually enforcing a complete blurring of boundaries; and, if there ever was any distance before, there is now complete overlap in my life — no more hyphen or separation in ‘work–family balance', and definitely no balance at all. I feel guilty for being so selfish in my unarticulated prayers focused on the wellbeing of my family, my ability to provide for them and others, and recognition for my efforts. There are more urgent needs, more disadvantaged environments, more paralysing tragedies. There are overcrowded living spaces, lack of basic hygiene, unaffordable health care, existing medical conditions, violent households, poverty and much more.

Regardless of this innate tendency to retreat back and isolate to protect myself and others near me, both physically and emotionally, I feel it is also my duty to become aware and understand other stories, other lives. At this time of social distancing, online socializations have become even more important through video phone calls made not only with relatives far away, but also with colleagues and friends from work who share virtual coffees with me, their insecurities and fears, their spaces and personal environments. It is comforting to find the testimonies of women I know and admire on Facebook — they are challenged, lost, hectic, paralysed, scared, hopeful and kind. I thank them for sharing themselves with me, the world. Our common weakness makes us stronger. Acts of kindness, often surprising, come frequently, in person and virtually — solidarity takes the form of email and Twitter messages in recognition of the work I am doing in my organization; colleagues asking after my family; senior managers sharing their anxieties and refusing to lead in a ‘masculine' way; consideration of how decisions will impact on diverse and disadvantaged groups of people; and the rejection of disembodied approaches to managing and organizing. Colleagues I barely know who live on their own offer to go out in search of nappies for my daughter; others send us messages acknowledging our lack of family in this area and offer support in case of illness. Our friendly neighbours' text offers of help with grocery shopping or other tasks; strangers around us create a group in support of those who are in need, disabled, self‐isolating or ill. After a string of political decisions and social behaviour that made me feel at risk and unwanted, my faith in the people around me is starting to grow back.

I know I have a huge amount of privilege compared to others. Amongst the many blessings, the first thing to note is the fairly sound health of my immediate family, although the circumstances of my parents and other relatives in Italy and Tanzania are a constant cause of worry. I won't be able to travel to say goodbye if they become ill; funerals have been banned in Italy and they will be alone in hospitals. Scenes of the months my mother spent in intensive care flash across my memory, I feel scorched in my core and helpless, and I try to push them away. I think of the many people here who are away from their loved ones, and our students who must feel vulnerable like I did during my postgraduate course in China at the time of the SARS outbreak.

My husband and I work at the same university, which is an advantage but could also become a single point of failure in case of potential future financial instability. I don't want to think of that eventuality. Staff have been told from the start of our communications regarding COVID‐19 that our wellbeing and our families are recognized priorities, so people who need to stay home due to caring responsibilities and self‐isolation will continue to be paid; capital investment has been put second to people's jobs. I am proud of the decisions we have taken and the calls we have made to protect our community so far. We were given tools and equipment to work from home, training is available and flexible support is plentiful, but that seems to be a rare occurrence in our industry. I see on social media many stories of people who are in the same sector and yet have no job safety, or others who have been fired and are desperately trying to find solutions to be able to take care of themselves and their families. Friends who are self‐employed and tradespeople report cancellations and lack of income. Only four days ago the Prime Minister asked people to remain home and imposed restrictions on movement. We don't know for how long this pandemic emergency may continue. This is an unprecedented level of insecurity and it makes managing and supporting others even more challenging.

Other forms of privilege I have are based on the structures and the socioeconomic matrix around me. I was able to take advantage of a wonderful nursery, which is now only open to children in families with key workers, and I will be able to save on fees during times of closure. I live in a country with public health provision, with good health standards and safety measures. We have food, which we try to buy and use responsibly even though others are stockpiling and raiding supermarkets.

Many people around the world are not so fortunate, and I think of them often. I hear their desperate calls for help, I see horrors in children's eyes that should have never materialized, and even more exacerbated tragedies for those who have suffered from wars, famine and abuse. Social awareness and civic conscience are now needed more than ever. Feminist thinking and community work are key to the development of empathy and actions that foster togetherness rather than otherness. So what can I do to help others? What can I do to move the needle towards a feminist way of thinking and living? It's important that I become proactive on this in many aspects of my personal and professional life. I draw up a list on my phone in the eerie silence of this pandemic lockdown night.

I am saddened that some people still think that Feminism is all about angry women who hate men. Surely, in 2020, people would have a more nuanced understanding of this after the many publications across disciplines that address feminist theory and approaches. I have come to think of Feminism as inextricably linked with equality, diversity and inclusion — terms that are more widely understood and embraced but not necessarily less complex. Although stemming from women's rights, to me Feminism is really about offering all people equal opportunities, regardless of their sex, gender, age, race, ability, class, background and other factors that make up their identities. It's about multi‐vocal pluri‐perspective conversations between individuals that challenge taken‐for‐granted structures and assumptions, which become collective projects linking the singular to the plural, the local to the systemic. To approach life with a feminist mind frame is to become (self)aware and (self)reflective about dynamics of power, privilege and discrimination at the individual, group and social level, with the aim to end oppression through equality for all. To think in a feminist way to me also means acknowledging and valuing difference; being mindful of intersectional issues; including voices, experiences and knowledge of women and others in every conversation and decision‐making process, at every level. As a result of this, Feminism is also inherently political in its challenges to the *status quo*, in its advocacy for equality and its promotion of values rooted in fairness, agency, humanity and interdependence. I think I have always been a feminist, even before I knew what it meant. I am proud to call myself a feminist, and I strive to work every day on myself and with others to become a better one.

‘*I want read book Mamy.*' She hasn't learned to use prepositions yet, it makes me smile, like her funny pronunciation of some words. I look at her little library of used books. I have tried to include a variety of little volumes portraying experiences of different cultures, perspectives and knowledge to expose her to a broad spectrum of humanity. I am proud of the fact that she loves reading. I am aware that my ability to provide for her with anything she many need to grow and develop is another one of the many privileges we have.

‘*I play towers now, ok*?' she suggests as a viable alternative to book reading in the middle of the night. I shake my head with a smile. I desperately want to be a good mother, teach her to be a good human being who is kind to others, see her become a young person making her own decisions about life. I need to wash my hair and look presentable for my meeting tomorrow, but I have been up for a while in the middle of the night now and I know I will prioritize sleep. I will also need to do the laundry, take out food for lunch, see if we can get a better deal on our bills now that we spend all our time at home using up heating, electricity, water, etc. I wonder if bad hair even shows on video. I should have invested in dry shampoo like my mommy friends suggested, now I can't go out to buy some. I forgot to get a birthday present for my daughter's little friend, but I guess we won't be attending any party now for the foreseeable future, so that's sorted. I need to send my mom some high‐quality masks; the nurse asked to check if I can find any online; the shipping to Italy will cost a fortune, but otherwise she will not survive this.

Last week, the first two days of working from home I had six and eight hours of virtual meetings, respectively. It feels like it's getting a bit better this week, but work at the moment is intensive and tiring. These meetings were urgent, strategic and encapsulated within a framework that had been developing over the previous four weeks: approving hundreds of alternative assessment methods in each of the seven departments I am responsible for; conceiving new courses that may attract students; re‐envisaging ways to teach and learn in this new university context; managing panic and stress from a number of staff; providing advice and reassurance; selling projects we ourselves may not be completely in agreement with. This pace of change and decision‐making is not sustainable, and I hope we will reach a point when things will be easier. Many of my colleagues only started coming to terms with the urgency and gravity of the situation last week, while I felt already exhausted after weeks of liaison with key role holders in departments and sections. I think given the role I chose to take on and the current circumstances, we'll have to just hang in there and plough through this for now. I don't have the luxury to stop; I have to work as efficiently as possible to make sure I support the organization in implementing the best plan we could design in order to address — and hopefully overcome — the unprecedented challenges brought by this pandemic.

My child wants to play now, even if it is in the middle of the night, and every day. She wants to build Duplo towers and read stories whilst her father and I try to alternate childcare and working from home. I wish that working from home was just responding to a few emails and reading books for my research. I haven't been able to work on my scholarly activity in over a month, and I miss it. I am not able to focus on my research now, as all work apart from COVID‐19 activity has been de‐prioritized. What will the effects be on my career? Am I selfish for thinking about my future at this time? Of course, this is the year when I decided to finally put in an application, and all promotions have been postponed. Hopefully not cancelled. Will I need to ask for an extension to deliver the book manuscript next year? What happens if my co‐author and I are asked to do further work this summer on the paper we submitted last month? Most of my books and resources are in my office, Internet services are slow and overwhelmed, and systems are limited. Even doing literature research for this piece seems an unsurmountable mountain, so I give up — maybe my own life tale will be enough.

We asked staff to switch to online delivery at light speed and, luckily, we only had one week of teaching left until the end of term. But assessment needs to go on to safeguard academic quality and standards, and some departments will teach after the Easter holidays, so measures must be developed, considered and approved in advance. I know that this has been extremely difficult for everyone, and people are working beyond what is expected, and sometimes what is reasonable. When we started to consider options a few weeks ago, most people were surprised but receptive; but we were also told that ‘university management' (as a disembodied abstract entity rather than a group of about 15 people we all know) was ‘over‐reacting', ‘it's just a flu' and ‘we won't come to a shut down, we are not Italy and China'. And yet, here we are — confined in our homes, relying on online delivery, having to learn to manage our lives and work in new ways.

The emotional labour involved in doing this work is unprecedented for me. My father pointed out that this type of activity is what I am best suited for, what I thrive in, the time where my best skills come out. He says I have always been particularly good at shifting into action and ‘going up a gear' in times of emergency or need, which is when I give the best of me. I wish it felt that way for me too. But I do feel somewhat proud, almost honoured I'd say, to be in a position whereby I can support others and make a difference, even if it is a challenging time. The work behind the scenes is invisible: weeks of endless emergency planning and contingency projections, of financial calculations, worry and care. With the current way that universities are funded in the UK, we simply cannot afford to close courses and not operate online. This is about survival.

This form of working from home and digitalization of tasks and relationships is extremely challenging. I see my husband delivering teaching and assessment online, supporting colleagues who have never had experience of this, having to catch up on knowledge and technology hardly ever heard of before, and balance it all off with his own studies, family life and childcare. If life commitments and work are two shifts in a woman's life, as articulated in the book by Arlie Russell Hochschild with Anne Machung, this new way of working in times of pandemic emergency feels like ‘The Never‐ending Shift'. Private homes are invaded through monitors; tiny cameras open up an immense window into our personal lives: our messy living rooms, the laundry hanging up in the kitchen, the pets needing limelight and children seeking undivided attention. I am very protective of my family space and I only want to share it selectively. Our sleep is often interrupted with worry and pain, our processes and habits are manipulated into something new that we do not recognize and yet need to adjust to at pace. This digital invasion is chipping off at the source of wellbeing my home offers in terms of comfort, protection and safety from the outside world. I wonder what my life looks like from the outside, from the other side of the camera lens. I am comforted by the opportunity to use a virtual backdrop in my online conversations, and yet I feel disturbed by it as it introduces a fake filter against the authenticity of my interpersonal connection.

‘*I no sleepy now. I hungry*' she declares sitting up. I avoid engaging further in what I know are tactics deployed to avoid going to sleep. What food do we have in the house? What will I be cooking tomorrow for dinner? Do I have time to make anything in between meetings? Maybe I can bake with my family after office hours, create memories. What if we won't have the opportunity to make those memories anymore, at some point, maybe soon? I need to keep my family well fed, with rationed yet nutritious meals. I realize that 95 per cent of our diet is based on fresh food — how long will we be able to have this? How many meals can I create with what I have in the house right now? I was so absorbed in back‐to‐back meetings today that I forgot to drink water, and then developed a headache, which made work even more exhausting. My back issues are now tormenting me, and the only way to avoid being crippled by pain is to work whilst sitting in bed, which may not be perceived by many as ‘professional enough', so on goes the fake room background. The incredibly fast‐paced rhythm of the past few weeks has demanded a very full schedule, which makes shopping for groceries problematic, as things like milk, eggs and other necessary items are only but memories on empty shelves adorned by meaningless price tags at the end of the day. Other countries have had shortages of toilet paper, hand sanitizers, medicines, masks and cleaning products, but here in the UK stockpiling of food has been a vicious outcome of this pandemic outbreak (even though this is barely the start of the ‘pandemic fight process' here). Many people are supporting others though, like the amazing Jack Monroe who is the author of cooking books for those who need to rely on canned food or very small budgets — I follow them on Twitter, and Jack is a continuous source of feminist inspiration.

‘*Mamy, are you happy*?' She has been asking this fairly regularly over the past two weeks. It caught me by surprise, and I felt a little broken inside, as a woman and as a mother. She can clearly feel my anxiety, even though I talk myself into thinking that I am fine, that I have things under control. ‘*Of course, my love. Mamy is very happy.*' She seems reassured, trusting as only young children can be at the unquestionable truth spoken by parents, and so she closes her eyes, with Grey Bunny safely tucked under her arm, her breath becoming slower and noisier. I collect the bottle of milk from under her sleeping bag, carefully plant a light kiss on her forehead and make my way back to our ‘big bed'. I wish I could just go back to sleep now, but my brain has gone into analytic overdrive and I start to consider assessment options, blended delivery plans, staff cover issues, student welfare and a million other matters. I think that I might as well have a look at my emails now, since I am awake; it will be fewer to do in the morning. Yesterday, I had almost 200 emails coming in during my first three hours of meetings; those are emails that require an action or an answer. I am on constant hyper‐performativity or speed‐dial mode, but I need to create pockets of normality within my work to ensure self‐care and foster wellbeing. Sometimes it's just easier to keep going, but I must be more disciplined with this. I grab my smartphone from the bedside table, quickly skipping the news notifications reporting on death tolls and escalation of safety measures. My husband's silent embrace reminds me that it is also up to me to create boundaries within this new work reality where the ‘never‐ending shift' seems to go uncontested. And so I make a promise to myself to think more effectively of new ways of living and organizing within the current circumstances, to create solutions that stem from feminist values in order to foster collective and individual approaches based on respect, solidarity and support. And feminist re‐actions to life in today's organizations, in this case specifically within the academic context, also include writing differently, honestly and instinctively about the emotional, embodied and contested experiences of people at work; we need stories that explore the current increasingly ambiguous space of ‘the workplace', to open up spaces of awareness, dialogue and togetherness.

## DECLARATION OF CONFLICTING INTERESTS

The author declared no potential conflicts of interests with respect to the authorship and/or publication of this article.

